# 6 The overlooked importance of normalising physical activity in cancer care

**DOI:** 10.1093/eurpub/ckae114.026

**Published:** 2024-09-26

**Authors:** Beth Brown

**Affiliations:** National Centre For Sport & Exercise Medicine, Sheffield, United Kingdom

## Abstract

**Background:**

Physical activity has a range of benefits for people diagnosed with cancer. Despite this, physical activity is not routinely promoted within healthcare and most people diagnosed with cancer aren’t active to recommended levels.

**Purpose:**

The project explores how a culture where physical activity is promoted as part of standard cancer care has been achieved in the USA and Canada. Insights can be used to improve the design and delivery of physical activity support.

**Methods:**

The Fellowship involved travelling to Canada and USA. Physical activity programmes that were integrated in clinical care using innovative approaches were identified. The project investigated how and why the programmes had become successful by observing operations and interviewing professionals. Differences between approaches in the USA, Canada and the UK were explored.

**Results:**

A range of cultural and contextual differences impact the extent to which physical activity is embedded as part of standard care. The prescriptiveness of programmes, and the delivery of exercise as medicine compared to physical activity was identified as a cultural difference that impacted on uptake and adherence. The integration of exercise facilities within healthcare settings helped to normalise physical activity for clinical staff and patients. Implementing systematic triage methods and utilising the workforce appropriately allows scalability. Funding from healthcare systems does not impact the success or sustainability of physical activity programmes.

**Conclusions:**

The marker of success within exemplar programmes was the normalisation of physical activity within a clinical system. The importance of design features that make physical activity look and feel like part of standard health care should not be overlooked.

**Practical implications:**

Recommendations have been created for decision makers designing cancer care and physical activity services. This includes how physical activity can be normalised by the design of environments, service pathways, language, and professional relationships.

